# Correlation of preoperative gut microbiota and postoperative delirium in patients undergoing minimally invasive direct coronary artery bypass grafting

**DOI:** 10.3389/fmicb.2026.1854098

**Published:** 2026-07-15

**Authors:** Ruiliang Hu, Yinglun Fang, Pengyang Han, Yongzheng Han, Jun Wang, Hongchao Liu, Yuning Shi, Zhengqian Li, Lei Zhang

**Affiliations:** 1 Department of Anesthesiology, Peking University Third Hospital, Beijing, China; 2 Beijing Center of Quality Control and Improvement on Clinical Anesthesia, Beijing, China; 3 Department of Laboratory Medicine, Peking University Third Hospital, Beijing, China; 4 International Medical Service, Peking University Third Hospital, Beijing, China

**Keywords:** bacterome, elderly individuals, gut microbiota, MIDCABG, mycobiome, POD

## Abstract

**Background:**

Postoperative delirium (POD) is a common complication after cardiac surgery, but the role of preoperative gut microbiota, especially the mycobiome, in POD susceptibility remains unclear. This study investigated the association between preoperative gut bacterome and mycobiome profiles and POD in elderly patients undergoing minimally invasive direct coronary artery bypass grafting (MIDCABG).

**Methods:**

Preoperative fecal samples from 31 elderly patients scheduled for MIDCABG were analyzed via 16S rRNA and ITS high-throughput sequencing. POD was assessed twice daily using the 3D-CAM scale. Patients were divided into delirium (M, *n* = 7) and control (C, *n* = 24) groups. Microbial diversity, composition, and bacteria–fungi correlations were systematically evaluated.

**Results:**

POD incidence was 22.58%. No baseline differences were observed between groups. Group M exhibited significantly lower gut microbiome health index and higher microbial dysbiosis index at both bacterome and mycobiome levels. Alpha/beta diversity did not differ significantly. LEfSe revealed bacterial enrichment of *Clostridium* and *Intestinibacter* in Group M, and *Bifidobacterium* in Group C. Fungally, *Aspergillus* and *Basidiomycota* predominated in Group M, while *Saccharomyces* and *Ascomycota* were enriched in Group *C. candida* showed the highest abundance in Group M and was a key node in the fungal co-occurrence network. Correlation analysis revealed positive associations between bacterial genera (*Klebsiella*, *Enterococcus*, *Roseburia*) and fungal taxa (*Wickerhamomyces*, *Pichia*, *Geotrichum*), suggesting ecological interactions potentially linked to POD.

**Conclusion:**

Preoperative dysbiosis of gut bacterome and mycobiome is associated with POD in elderly MIDCABG patients. Several bacterial and fungal taxa, including *Clostridium*, *Intestinibacter*, *Aspergillus*, *Saccharomyces*, and *Candida*, were identified as candidate microbial biomarkers associated with POD and may contribute to future microbiota-based risk stratification strategies.

## 1 Introduction

Postoperative delirium (POD) is an acute and transient brain dysfunction, commonly observed in patients over 60 years old within one week after cardiac surgery, with an incidence rate ranging from 3 to 55% (Katznelson et al., 2009; Staicu et al., 2025; Yang et al., 2024). It is characterized by attention deficits, altered levels of consciousness, and cognitive dysfunction (Huo et al., 2025; Deschamps et al., 2024). The occurrence of POD in postcardiac surgery patients often indicates an increased risk of poor prognosis, including a multiplicative increase in ICU stay time and total hospitalization duration, deterioration of cognitive function, higher medical costs, and more postoperative complications, even increased mortality (Cai et al., 2022). 96% of anesthesiologists in China recognize the importance of POD, but most rely solely on clinical observation for evaluation. Currently, there is still a lack of systematic approaches for assessment, monitoring, prediction, validation, and treatment (Delp et al., 2020; Yuan et al., 2022).

The gut microbiota accounts for 95% of the total human microbiota (Zhang et al., 2023). Previous studies have indicated that the total bacterome load in the gut decreases after cardiac surgery, with the impairment potentially persisting for over a week. Additionally, patients experiencing visual hallucinations exhibit increased levels of *Staphylococcus* and *Pseudomonas* in the gut, which are associated with POD symptoms (Maekawa et al., 2020). Another study on the gut microbiota of patients undergoing non-stop coronary artery bypass grafting found an increase in *Enterococcus*, an opportunistic pathogen, in the POD group (Huang et al., 2025). These findings suggest that alterations in the gut microbiota of cardiac surgery patients may be related to POD. Actually, bacterome is not the only microorganism residing in the gastrointestinal tract of humans in physiological conditions, because also mycobiome can be present, but its role in POD is far less known (Ticinesi et al., 2023). However, previous studies sequenced only the 16S rRNA gene for the bacterome group and did not analyze the mycobiome. With advancements in medical technology and the development of the Enhanced Recovery After Surgery (ERAS) concept, an increasing number of patients opt for minimally invasive direct coronary artery bypass (MIDCABG). MIDCABG advances surgical technology while offering substantial organ protection. Optimized anesthesia, for example through regional techniques and reduced opioid use, together with a less invasive surgical transition from conventional cardiopulmonary bypass to MIDCABG, effectively attenuates surgical stress on the intestinal microbiome (Wang, 2026b; Shi et al., 2025). Maintaining gut barrier function in this manner may in turn block the pathological sequence linking microbial dysbiosis to systemic and neuroinflammatory responses that culminate in POD. However, current research on the preoperative gut bacterome and mycobiome on POD in such surgical patients remains a gap.

Therefore, this study aims to investigate the preoperative gut microbiota diversity at both bacterome and mycobiome levels and the correlation with POD in elderly patients scheduled for MIDCABG, and to develop novel prevention and treatment strategies to mitigate its effects on peri-anesthetic recovery.

## 2 Materials and methods

### 2.1 Research design

This study is a single-center, cross-sectional study that enrolled 31 elderly patients scheduled for MIDCABG in the Department of Cardiac Surgery at Peking University Third Hospital between May 2025 and November 2025 (China Clinical Trial Registry number: ChiCTR2500101667). The study was approved by the Hospital Ethics Committee of Peking University Third Hospital (Approval No.: M20250188), and all procedures complied with the Declaration of Helsinki. All participants signed informed consent forms before enrollment. The study adhered to the Strengthening the Reporting of Observational Studies in Epidemiology (STROBE) guidelines. The inclusion criteria specifically included the following three items: (1) Age ≥60 years; (2) American Society of Anesthesiologists (ASA) physical status class III; (3) Patients scheduled for MIDCABG. The exclusion criteria included the following conditions: (1) Refusal to participate in the study; (2) Participation in other clinical trials; (3) Inability to complete preoperative evaluation due to severe dementia, language impairment, severe hearing/visual impairment, coma, or terminal illness; (4) Preoperative screening revealed cognitive impairment; (5) Use of probiotics, prebiotics, or other microbial preparations within the past 3 months; (6) Acute diarrhea within the past month.

### 2.2 Delirium assessment

Five days before surgery or before discharge, when the patient is awake, a professionally trained anesthesiologist administers the Chinese version of the “Three-Minute Diagnostic Assessment of Impaired Consciousness” (3D-CAM) for POD evaluation twice daily (7:00–11:00 and 18:00–22:00). This scale is widely used in the assessment of delirium in elderly patients and includes four characteristics: (1) acute onset with fluctuation; (2) inattention; (3) confusion; (4) altered level of consciousness (Ho et al., 2021; Shang et al., 2024). Delirium is diagnosed when both criteria 1 and 2 are met, along with at least one of criteria 3 or 4 (Yang et al., 2022). The type of delirium is further determined using the RAAS scale. All assessment results are documented, and a panel of three psychiatric experts reviews the diagnosis. In cases of uncertainty, a collaborative review process is conducted, requiring five rounds of deliberation. Long-term cognitive function is assessed by telephone at 1, 6, and 12 months postoperatively.

### 2.3 Anesthesia management

Upon entering the operating room, each patient received the same anesthesia protocol. Routine monitoring included continuous electrocardiogram, pulse oximeter, non-invasive blood pressure monitoring, and end-tidal carbon dioxide monitoring. BIS dual-frequency index monitoring (Suzhou Medion Life Technology Co., Ltd., China) was fixed on the patient’s forehead before anesthesia induction. Arterial catheters and central venous catheters were also placed before induction for invasive arterial blood pressure and central venous pressure monitoring. Induction was performed with sufentanil 0.5–1 μg/kg, etomidate 1.5–2 mg/kg, propofol 1.5–2 mg/kg, and cisatracurium 0.2 mg/kg. After tracheal intubation, mechanical ventilation with 60% oxygen was provided. The tidal volume was adjusted based on blood gas analysis to maintain normal arterial carbon dioxide levels. Anesthesia depth was controlled by adjusting the inhaled sevoflurane concentration by 1–2% based on hemodynamic response and BIS values (target range: 40–60). Sufentanil 10–20 μg and cisatracurium 2–4 mg were administered every hour to maintain anesthesia depth. If MABP < 70 mmHg, the patient was immediately treated with norepinephrine or epinephrine to ensure MABP > 70 mmHg. When HR < 50 bpm, cardiovascular active drugs such as anisodamine were used (avoiding anticholinergic drugs). Cefuroxime sodium was administered 30–60 min before surgery to prevent infection. All patients underwent MIDCABG under the guidance of a designated surgeon. Postoperatively, a 1% ropivacaine 250 mL intercostal nerve analgesia pump was routinely used, and the patient was transferred to the ICU monitoring ward with a tracheostomy tube. The dosage would be adjusted according to individual conditions until an adequate analgesic effect was achieved.

### 2.4 Biological sample collection

Preoperative stool samples were collected in sterile tubes at the time of patient admission (not part of preoperative bowel preparation). Fresh stool samples (0.5 g) were collected from each participant, placed in sterile containers, and immediately stored at −80 °C for further processing. According to the manufacturer’s instructions, genomic DNA was extracted from the stool samples using the Stool DNA Isolation Kit (Foregene, China).

### 2.5 Extraction of fecal DNA and PCR amplification

After genomic DNA extraction, DNA concentration and purity were determined. For bacterome amplification, the primers 338F (5'-ACTCCTACGGGAGGCAGCAG-3') and 806R (5'-GGACTACHVGGGTWTCTAAT-3') were used to amplify the bacterome region 338F_806R. For mycobiome amplification, the primers ITS1F (5'-CTTGGTCATTTAGAGGAAGTAA-3') and ITS2R (5'-GCTGCGTTCTTCATCGATGC-3') were used to amplify the mycobiome region ITS1F_ITS2R. All PCR reactions were performed using Pro Taq with a 20 μL reaction mixture: 10 μL of 2 × Pro Taq buffer, 0.8 μL of upstream primer (5 μM), 0.8 μL of downstream primer (5 μM), and 10 ng of DNA sample, supplemented with sterile enzyme-free water to 20 μL.

### 2.6 High-throughput sequencing analysis of bacterome 16S rRNA gene and mycobiome ITS gene

High-throughput sequencing analysis of the bacterome 16S rRNA gene and the mycobiome ITS gene was performed using the Illumina NextSeq 2000 system at Shanghai Meiji Biopharmaceutical Technology Co., Ltd. After completing genomic DNA extraction, 1% agarose gel electrophoresis is used to detect the extracted genomic DNA. Genomic DNA meeting detection criteria was processed to construct PE libraries, followed by fragment screening, enrichment, and denaturation to obtain single-stranded DNA fragments. Fluorescence signal results collected in each cycle were statistically analyzed to determine the sequence of template DNA fragments. After sample demultiplexing of the sequencing-obtained paired-end reads, quality control and filtering are first performed on the paired-end reads based on sequencing quality. Meanwhile, the overlapping relationships between paired-end reads are used for sequence assembly to obtain optimized data after quality control and assembly. Sequence denoising methods were applied to optimize the data, yielding Amplicon Sequence Variant (ASV) representative sequences and abundance information. Based on ASV representative sequences and abundance information, a series of statistical or visual analyses were conducted, including species taxonomy analysis, community diversity analysis, species divergence analysis, correlation analysis, phylogenetic analysis, and functional prediction analysis.

### 2.7 Sample size calculation

Bayesian methods demonstrate significant advantages in small-sample studies by fully utilizing prior information and combining it with observational data for inference, thereby providing more robust results. Based on the Bayesian formula, we selected the Beta distribution as the prior distribution for the incidence rate p. $\begin{array}{l} \text{Beta}(\alpha \text{post},\;\beta \text{post})=\text{Beta}(\alpha \text{prior}+\text{successes},\;\beta \text{prior}+\text{failures}),\;\\ \mu \text{post}=\frac{\alpha \text{post}}{\alpha \text{post}+\beta \text{post}\;},\sigma \text{post}\\ =\sqrt{\frac{\alpha \text{post}*\beta \text{post}}{{\left(\alpha \text{post}+\beta \text{post}\right)}^{2}(\alpha \text{post}+\beta \text{post}+1)}},\;\text{and}\;n=\frac{Z^{2}*p(1-p)}{E^{2}} \end{array}$ is particularly suitable for handling probability parameters. According to previous literature (Katznelson et al., 2009; Staicu et al., 2025; Yang et al., 2024), with a POD incidence rate of 20%, we chose Beta (2,8) as the prior distribution and calculated the posterior distribution of the incidence rate as Beta (8,32). The mean of this posterior distribution was 0.20, with a standard deviation of 0.065, indicating that our estimate of the incidence rate falls within a reasonable range and has low uncertainty. Therefore, based on the calculation, Z is the *Z*-value of the 95% confidence interval (approximately 1.96), which necessitates the inclusion of 30 MIDCABG patients. Considering the attrition rate and other factors (calculated at 20%), this study plans to enroll 36 patients.

### 2.8 Statistical analysis

Descriptive statistics were used to summarize continuous variables. Data with normal distribution were expressed as mean ± standard deviation (SD), while non-normal data were presented as median and interquartile range (IQR). Categorical variables were reported as frequency and percentage. For intergroup comparisons, an independent-samples t-test was used for normally distributed variables, and a Mann–Whitney U test was used for non-normally distributed variables. A two-tailed *p* < 0.05 was considered statistically significant. All statistical analyses were performed using R software (version 4.5.2).

## 3 Results

### 3.1 General characteristics

A total of 31 patients were ultimately enrolled in this study and further divided into the delirium group (M group, *n* = 7) and the control group (C group, *n* = 24) based on the occurrence of POD. No significant differences were observed between the two groups in continuous variables such as age, preoperative albumin, postoperative albumin, operative duration, anesthesia duration, sufentanil dosage, and ICU stay time. Additionally, there were no significant differences in gender distribution, first postoperative bowel movement time, and educational level between the two groups (Table 1).

**Table 1:** Patient demographics and baseline characteristics.

Characteristic (mean ± SD, %)	Normal (C group) *N* = 24	Pod (M group) *N* = 7	*p*-value
Age (year)	67 ± 7	68 ± 11	0.911^1^
Preoperative albumin (g/L)	39.2 ± 4.0	38.5 ± 2.9	0.622^1^
Postoperative albumin (g/L)	32.38 ± 3.08	31.97 ± 3.27	0.775^1^
Operation duration (min)	210 ± 78	216 ± 57	0.842^1^
Anesthesia duration (min)	301 ± 84	318 ± 71	0.604^1^
Sufentanil used (μg)	130 ± 16	129 ± 17	0.876^1^
ICU duration (h)	34 ± 24	41 ± 42	0.658^1^
Gender			0.379^2^
Female	9 (37.5%)	1 (14.3%)	
Male	15 (62.5%)	6 (85.7%)	
Defecation time			>0.999^2^
1–5 days	19 (79.2%)	6 (85.7%)	
More than 5 days	5 (20.8%)	1 (14.3%)	
Education			>0.999^2^
Primary and junior high schools	15 (62.5%)	5 (71.4%)	
Senior high school	7 (29.2%)	2 (28.6%)	
College	2 (8.3%)	0 (0.0%)	

^1^Welch Two Sample *t*-test, ^2^Fisher’s exact test.

### 3.2 Alpha and beta diversity analysis of bacterial 16S rRNA gene

**Figure 1:**
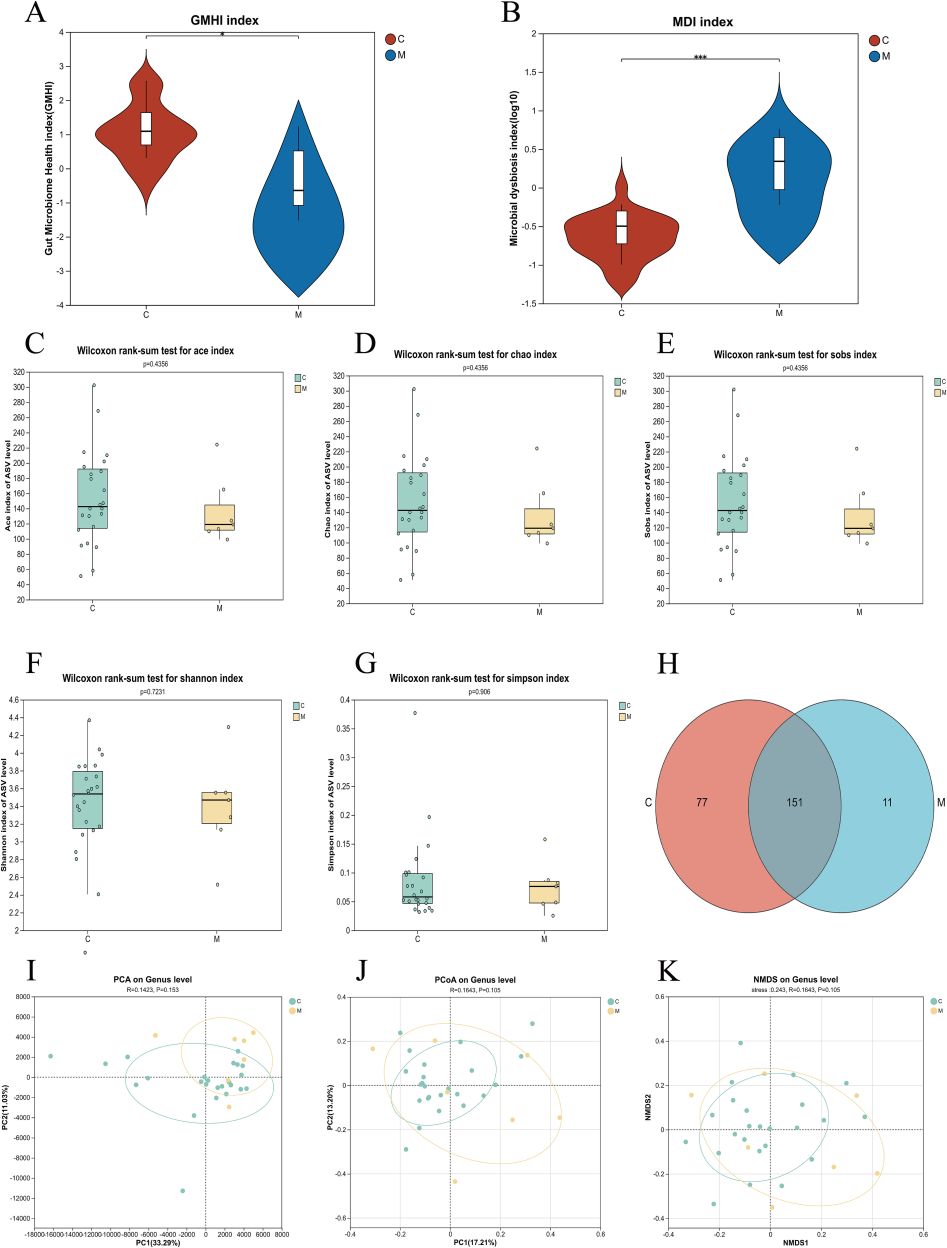
Alpha- and beta-diversity analyses of the preoperative gut bacteriome based on 16S rRNA gene sequencing. **(A)** Gut Microbiome Health Index (GMHI). **(B)** Microbial Dysbiosis Index (MDI). **(C)** ACE index. **(D)** Chao1 index. **(E)** Sobs index. **(F)** Shannon index. **(G)** Simpson index. **(H)** Venn diagram showing shared and unique bacterial ASVs between groups. **(I)** Principal component analysis (PCA). **(J)** Principal coordinates analysis (PCoA). **(K)** Non-metric multidimensional scaling (NMDS). *p < 0.05, ***p < 0.001.

High-throughput sequencing of the bacterome 16S rRNA gene was performed to analyze changes in the gut bacterome in fecal samples from 31 patients. At the ASV level, the gut microbiota health index (GMHI) in Group C was significantly higher than that in Group M; whereas the microbiota dysbiosis index (MDI) in Group M was significantly higher than that in the Group C (Figures 1A,B). Alpha diversity analysis between the two groups showed no significant differences in the ACE, Chao, Sobs, Shannon, and Simpson indices (Figures 1C–G). Additionally, principal component analysis (PCA), principal coordinate analysis (PCoA), and non-metric multidimensional scaling (NMDS) analyses indicated no significant differences in beta diversity between the two groups (Figures 1I–K). Venn diagrams revealed distinct microbial community structures in the two groups, comprising 151 species, with 77 species exclusive to Group C and 11 to Group M (Figure 1H).

### 3.3 Analysis of the composition of the gut bacterome

**Figure 2:**
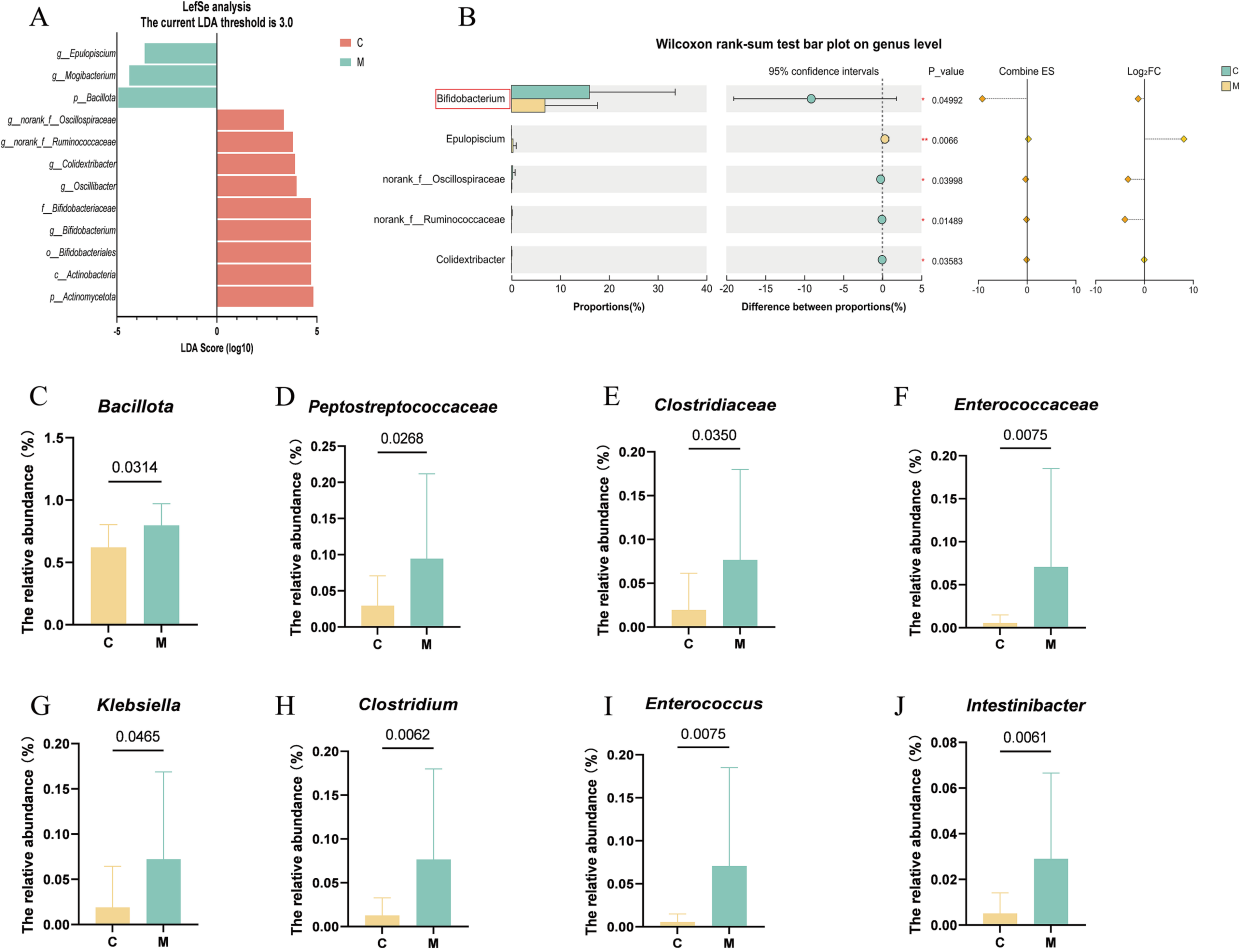
Differential composition analysis of the preoperative gut bacteriome. **(A)** Linear discriminant analysis effect size (LEfSe) analysis (LDA score > 3.0). **(B)** Differential abundance analysis at the genus level using the Wilcoxon rank-sum test. **(C)** Relative abundance of *Bacillota* at the phylum level. **(D–F)** Relative abundances of *Peptostreptococcaceae*, *Clostridiaceae*, and *Enterococcaceae* at the family level. **(G–J)** Relative abundances of *Klebsiella*, *Clostridium*, *Enterococcus*, and *Intestinibacter* at the genus level. *p < 0.05, **p < 0.01.

To further investigate the effects of different microbial communities, we generated linear discriminant analysis (LDA) score plots using LEfSe (Figure 2A). By setting the LDA threshold above 3.0, we determined the abundance distribution of microorganisms in each community. In the comparison between Group C and Group M, the predominant communities in Group M included *p__Bacillota*, *g__Mogibacterium*, and *p__Bacillota*, while those in Group C comprised *g__norank_f__Oscillospiraceae*, *g__norank_f__Ruminococcaceae*, *g__Colidextribacter*, *g__Oscillibacter*, *f__Bifidobacteriaceae*, *g__Bifidobacterium*, *o__Bifidobacteromees*, *c__Actinobacteria*, and *p__Actinomycetota*. Concurrently, comparative analysis using species and differential functional analyses revealed that *Bifidobacterium* exhibited higher abundance in Group C than in Group M (Figure 2B).

To investigate the specific changes in the gut microbiota, we analyzed alterations at the phylum, order, and genus levels. At the phylum level, the relative abundance of *Bacillota* in Group M was significantly higher than that in Group C (Figure 2C). Additionally, at the order level, the relative abundances of *Peptostreptococcaceae*, *Clostridiaceae*, and *Enterococcaceae* were significantly increased in Group M compared to Group C (Figures 2D–F). At the genus level, the relative abundances of *Klebsiella*, *Clostridium*, *Enterococcus*, and *Intestinibacter* were significantly increased in Group M (Figures 2G–J).

### 3.4 Co-occurrence network analysis and KEGG functional prediction of the gut bacterome

**Figure 3:**
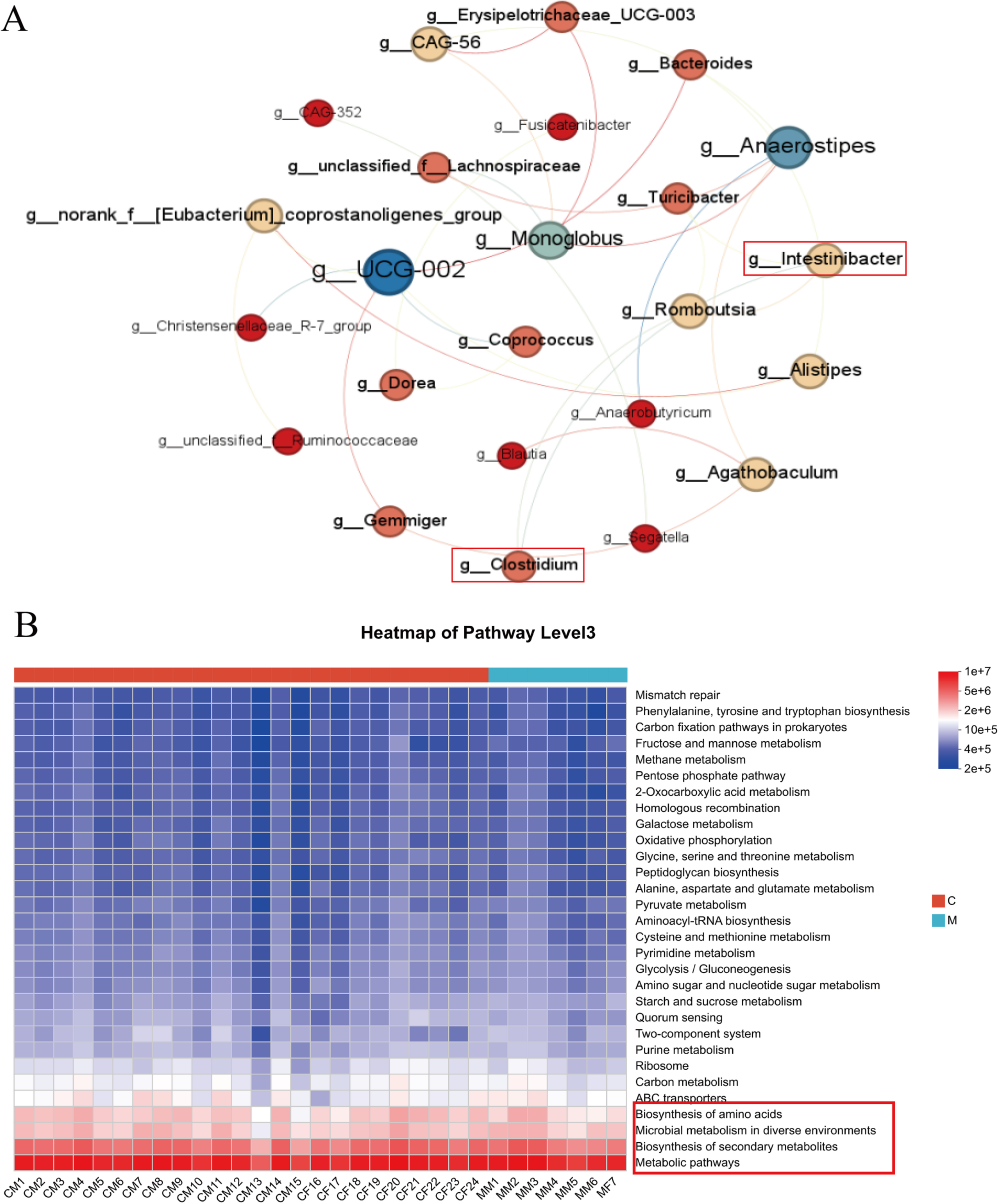
Co-occurrence network analysis and functional prediction of the gut bacteriome. **(A)** Bacterial co-occurrence network constructed at the genus level. Node size represents relative abundance, and edges indicate significant correlations between taxa. **(B)** Kyoto Encyclopedia of Genes and Genomes (KEGG) level 3 pathway prediction.

A co-occurrence network analysis of the top 50 bacterial genera revealed a complex interaction pattern within the gut bacteriome (Figure 3A). Several genera, including *UCG-002*, *Anaerostipes*, *Monoglobus*, and *Coprococcus*, occupied central positions in the network and exhibited multiple correlations with neighboring taxa. Notably, *Clostridium* and *Intestinibacter*, which were enriched in the POD group, were integrated into the bacterial interaction network and displayed correlations with several other genera, suggesting their potential involvement in the altered microbial ecosystem associated with POD.

KEGG level 3 functional prediction analysis demonstrated similar functional profiles between groups (Figure 3B). The most abundant predicted pathways included Metabolic pathways, Biosynthesis of secondary metabolites, Microbial metabolism in diverse environments, and Biosynthesis of amino acids, indicating that these metabolic functions represented the dominant functional characteristics of the gut bacteriome in both groups.

### 3.5 Alpha and beta diversity analysis of the mycobiome ITS region

**Figure 4:**
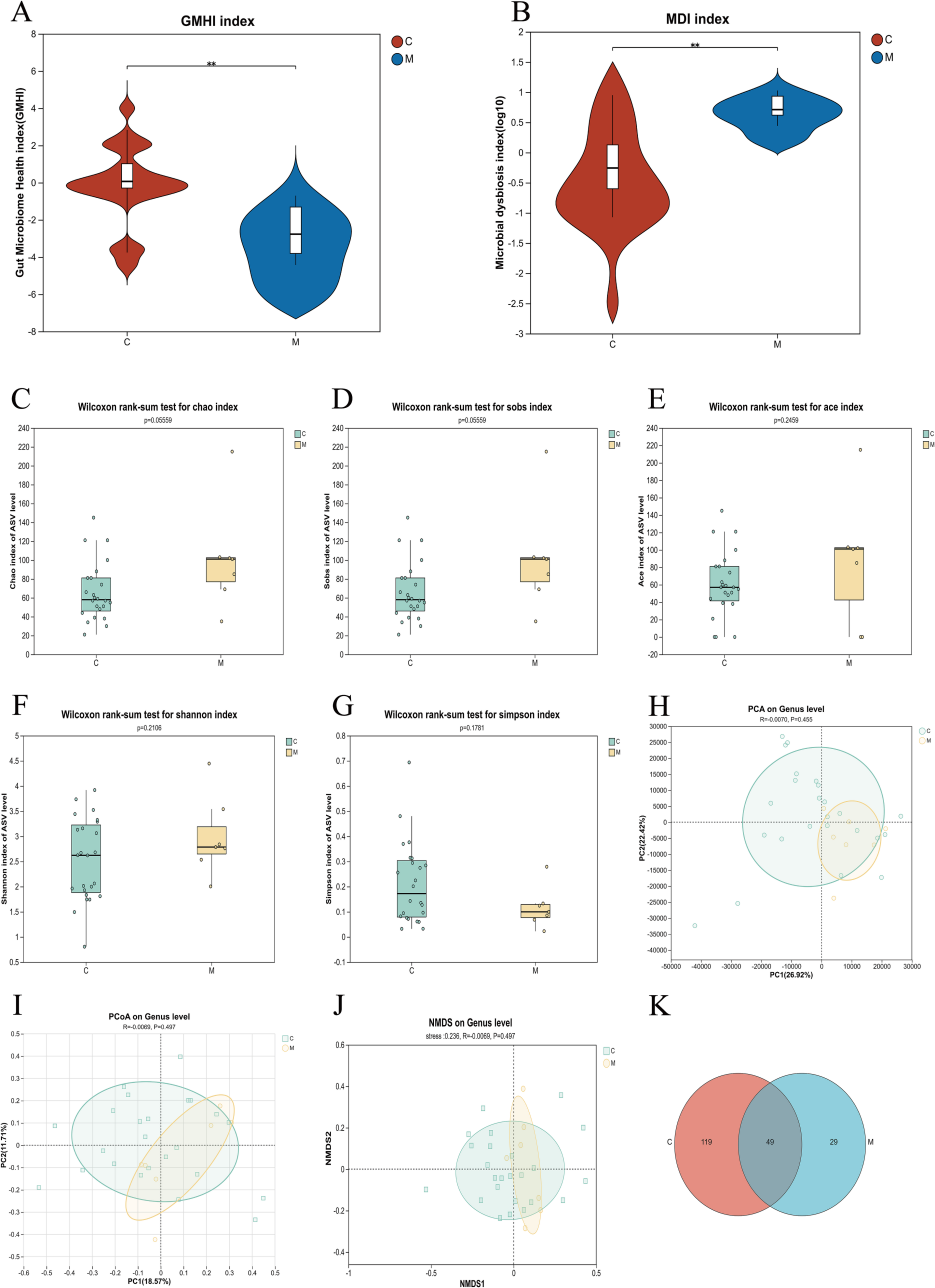
Alpha- and beta-diversity analyses of the preoperative gut mycobiome based on ITS region. **(A)** GMHI. **(B)** MDI. **(C)** Chao1 index. **(D)** Sobs index. **(E)** ACE index. **(F)** Shannon index. **(G)** Simpson index. **(H)** PCA. **(I)** PCoA. **(J)** NMDS. **(K)** Venn diagram showing shared and unique fungal ASVs between groups. **p < 0.01.

High-throughput ITS sequencing was performed to analyze changes in the gut mycobiome in fecal samples from 31 patients. At the ASV level, the GMHI of Group C was significantly higher than that of Group M, while the MDI of Group M was significantly higher than that of the Group C (Figures 4A,B). Alpha diversity analysis between the two groups showed no significant differences in Chao, Sobs, ACE, Shannon, and Simpson indices (Figures 4C–G). Additionally, PCA, PCoA, and NMDS analyses indicated no significant differences in beta diversity between the two groups (Figures 4H–J). Venn diagrams revealed distinct mycobiome community structures between the two groups, with a total of 148 species identified, 119 exclusive to Group C, and 29 exclusive to Group M (Figure 4K).

### 3.6 Analysis of the composition of gut mycobiome

**Figure 5:**
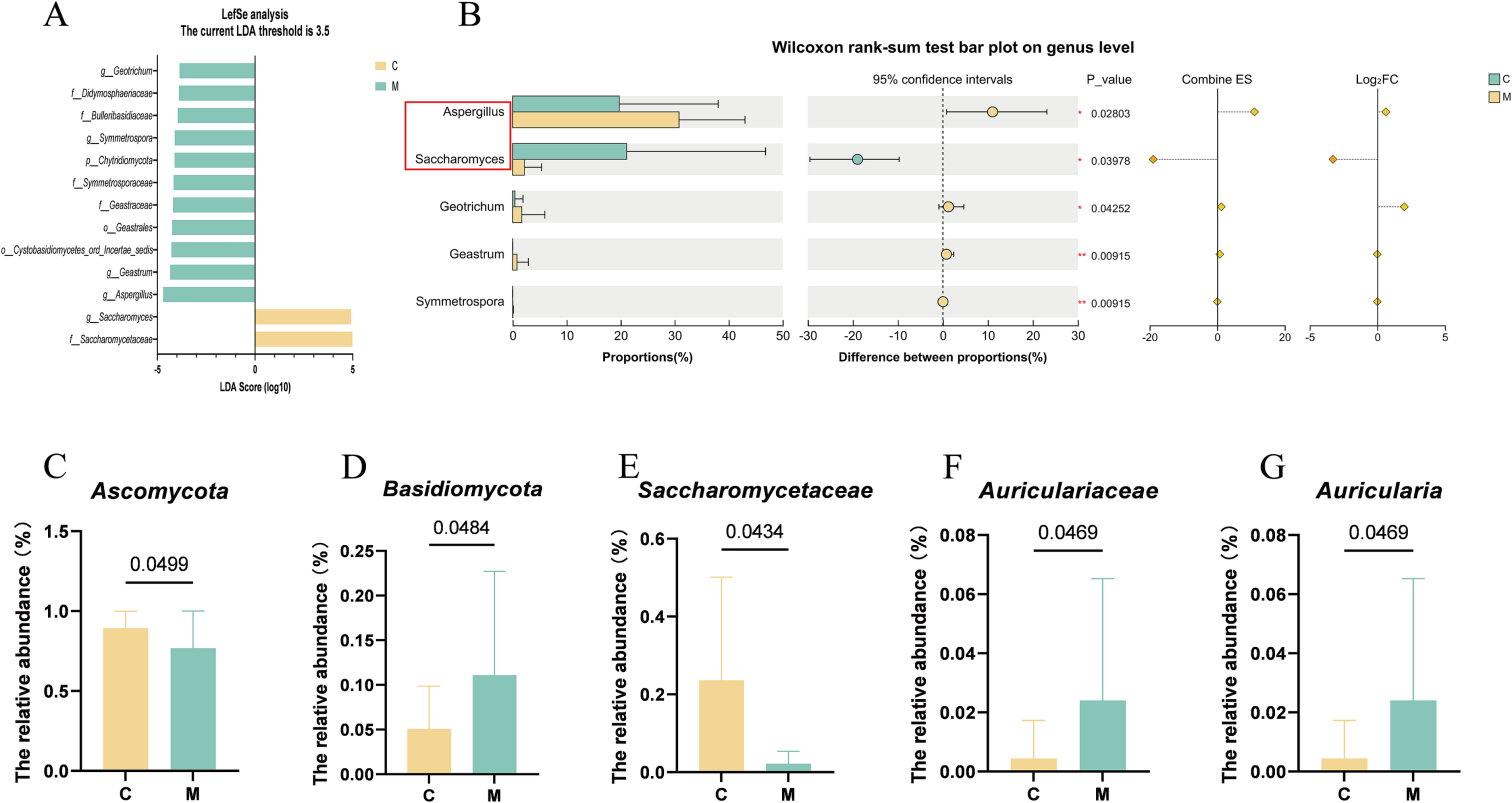
Differential composition analysis of the preoperative gut mycobiome. **(A)** LEfSe analysis (LDA score > 3.5). **(B)** Differential abundance analysis at the genus level using the Wilcoxon rank-sum test. **(C,D)** Relative abundances of *Ascomycota* and *Basidiomycota* at the phylum level. **(E,F)** Relative abundances of *Saccharomycetaceae* and *Auriculariaceae* at the family level. **(G)** Relative abundance of *Auricularia* at the genus level. *p < 0.05, **p < 0.01.

To further investigate the effects of different mycobiota, we performed LEfSe analysis and generated LDA score plots (Figure 5A). By setting the LDA threshold above 3.0, we determined the abundance distribution of microorganisms in each community. In the comparison between Group C and Group M, the predominant communities in Group C included *f__Saccharomycetaceae* and *g__Saccharomyces*, while those in Group M comprised *g__Aspergillus*, *g__Geastrum*, *o__Cystobasidiomycetes_ord_Incertae_sedis*, *o__Geastrales*, *f__Geastraceae*, *f__Symmetrosporaceae*, *p__Chytridiomycota*, *g__Symmetrospora*, *f__Bulleribasidiaceae*, *f__Didymosphaeriaceae*, and *g__Geotrichum*. Concurrently, comparative analysis using species and differential functional analyses revealed a higher abundance of *Aspergillus* in Group M and of *Saccharomyces* in Group C (Figure 5B).

To investigate the specific changes in the gut mycobiota, we analyzed alterations at the phylum, family, and genus levels. At the phylum level, the relative abundance of *Ascomycota* was significantly higher in Group C than in Group M; conversely, the relative abundance of *Basidiomycota* was significantly higher in Group M than in Group C (Figures 5C,D). Additionally, at the family level, the relative abundance of *Saccharomycetaceae* was significantly reduced in Group M compared to Group C, while the relative abundance of *Auriculariaceae* was significantly increased in Group M (Figures 5E,F). At the genus level, the relative abundance of *Auricularia* was significantly increased in Group M (Figure 5G).

### 3.7 Co-occurrence network analysis of gut mycobiome

**Figure 6:**
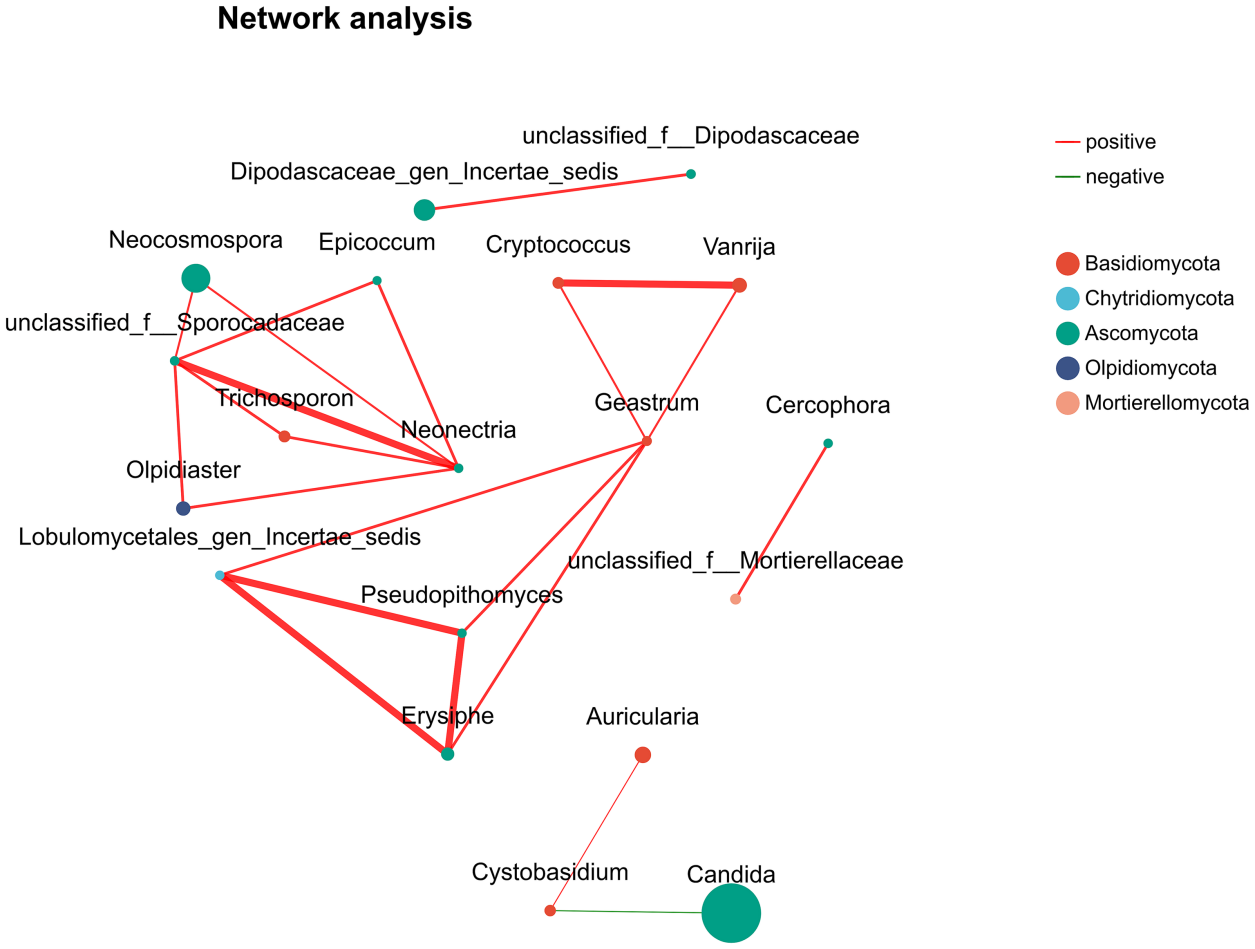
Co-occurrence network analysis of the gut mycobiome. A fungal co-occurrence network constructed using the top fungal taxa at the genus level. Node size represents relative abundance, and edges indicate significant correlations between fungal taxa.

A co-occurrence network analysis was performed using the top 50 fungal taxa to characterize potential interactions within the gut mycobiome (Figure 6). The network was predominantly composed of positive correlations, whereas only a limited number of negative associations were observed. Several fungal genera, including *Geastrum*, *Neocosmospora*, *Olpidiaster*, *Epicoccum*, *Cryptococcus*, and *Vanrija*, formed a tightly connected interaction network, suggesting potential ecological cooperation among these taxa. Among the identified fungi, *Geastrum* occupied a central position within the network and displayed multiple connections with neighboring taxa, indicating a potentially important role in maintaining fungal community structure. In contrast, *Candida* exhibited the highest relative abundance, as reflected by its large node size, but showed no significant co-occurrence relationships with other fungal taxa, suggesting that its enrichment may occur independently of the major fungal interaction network. These findings indicate that the fungal communities associated with POD are characterized not only by alterations in taxonomic abundance but also by changes in inter-fungal ecological relationships.

### 3.8 Bacterome-mycobiome correlation analysis

**Figure 7:**
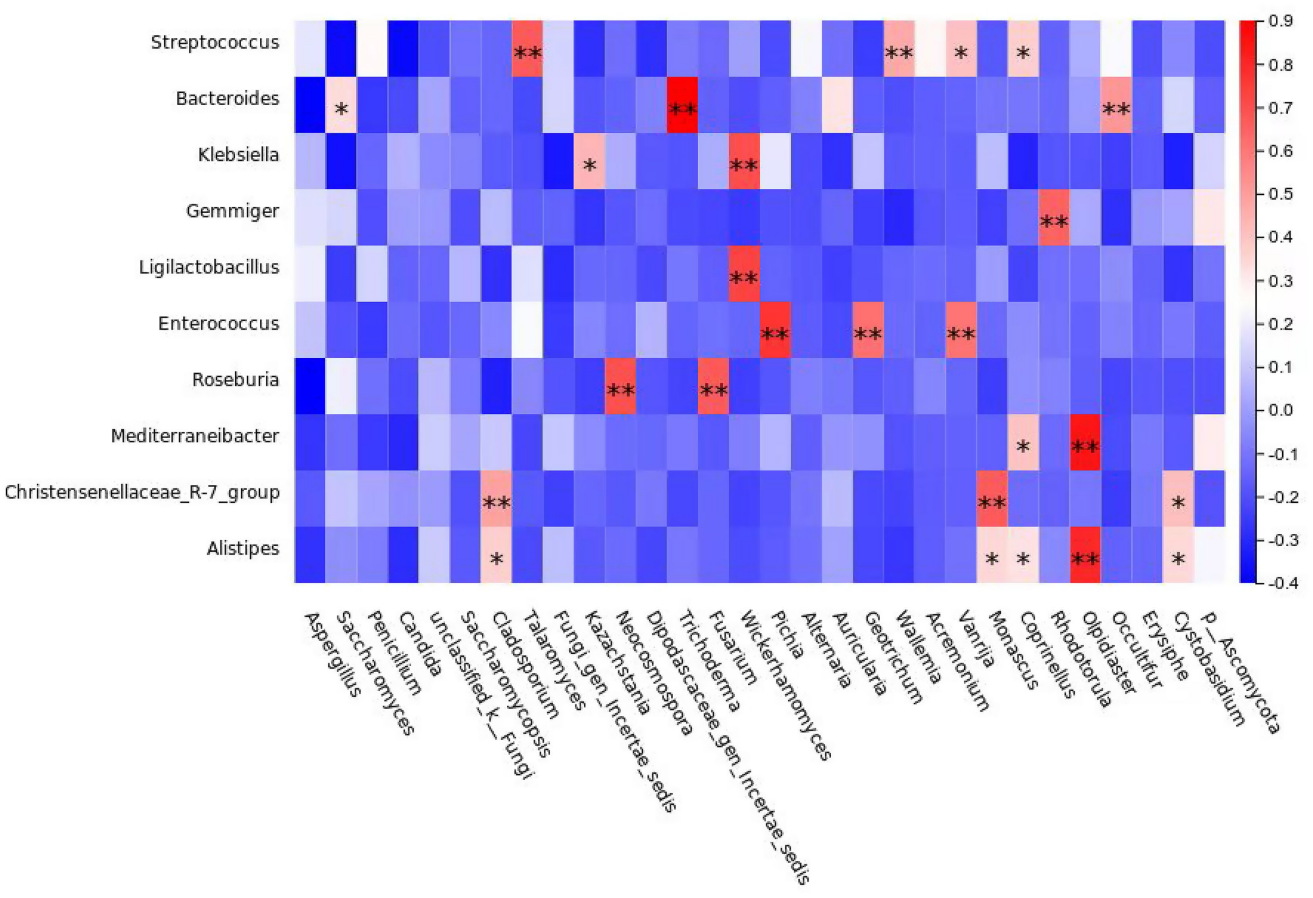
Correlation analysis between the gut bacteriome and mycobiome. Heatmap showing significant correlations between bacterial and fungal genera. Correlation coefficients were calculated using Spearman’s rank correlation analysis. Positive and negative correlations are indicated by the color scale, and significance levels are denoted by asterisks. *p < 0.05, **p < 0.01.

Correlation analysis revealed significant bacteria–fungi interactions within the gut microbiota (Figure 7). *Klebsiella*, *Enterococcus*, and *Roseburia* exhibited significant positive correlations with multiple fungal taxa, including *Wickerhamomyces*, *Pichia*, *Geotrichum*, *Vanrija*, *Kazachstania*, and *Fusarium*. Furthermore, *Mediterraneibacter* and *Alistipes* were positively associated with *Olpidiaster*. These results indicate potential ecological interactions between bacterial and fungal communities within the gut microbiota of patients undergoing MIDCABG.

## 4 Discussion

No statistically significant differences in baseline characteristics were observed between the two groups in our study. The incidence of POD was 22.58%, consistent with rates reported in recent related studies (Yang et al., 2024; Staicu et al., 2025). Compared with Group C, Group M exhibited a less favorable preoperative gut microbial profile, characterized by lower GMHI, higher MDI, and distinct alterations in both bacterial and fungal community composition. GMHI is a composite metric developed to quantify the degree of microbiome health based on the relative abundance of health-associated and disease-associated microbial taxa. Higher GMHI values generally indicate a microbiota composition more frequently observed in healthy individuals. In contrast, MDI reflects deviation from a balanced microbial ecosystem, with higher values indicating greater dysbiosis (Chang et al., 2024; Zielińska et al., 2025; Ma et al., 2025). Therefore, the concurrent reduction in GMHI and elevation in MDI observed in the POD group suggest a less favorable preoperative intestinal microbial profile. Preoperative multimodal phenotypes, including gut microbiota diversity, short-chain fatty acids (SCFAs) levels, inflammatory factors, and circadian rhythms, have been identified as robust predictors of POD and long-term cognitive outcomes in colorectal cancer patients (Pan and Wang, 2026). Likewise, the preoperative detection of *Shigella* has been shown to predict POD following abdominal surgery (Liu et al., 2022). The baseline differences observed between the two groups in the present study are consistent with these previous findings. However, previous research has generally suggested that multiple perioperative factors, such as fasting, antibiotic use, opioids, and surgical stress itself, severely disrupt the intestinal microbial ecosystem and exacerbate the incidence of POD (Wang, 2026a; Shi et al., 2025). Our results indicate that POD patients exhibit a susceptible intestinal microbiota state before MIDCABG surgery, suggesting that the abnormality of intestinal microbiota may precede POD occurrence.

The enrichment of *Clostridium* species in this study is particularly noteworthy, with findings largely consistent with those reported in previous study (Cao et al., 2026). *Clostridium* is a highly heterogeneous genus that includes various opportunistic pathogens, such as *Clostridium difficile*, whose infection is closely associated with severe intestinal inflammation and systemic complications (Yang et al., 2026). A case report of fecal microbiota transplantation in an 92 year-old subject with recurrent *Clostridium difficile* infection also showed that the procedure determined complete recovery of persistent delirium (Gotoh et al., 2022). Abnormal increases or decreases in *Clostridium* species are often correlated with other disease states. In schizophrenia patients, *Clostridium* species are significantly enriched in the gut microbiota and are associated with inflammatory states (Xu et al., 2025); in patients with relapsed or refractory lymphoma, the abundance of *Clostridium* species is reduced (Guo et al., 2026). These results reflect the functional heterogeneity of different *Clostridium* species and their complex associations with specific disease backgrounds, which may involve pro-inflammatory or neuroactive substance-producing capabilities, thereby contributing to the pathogenesis of POD.

The enrichment of another key bacterome genus, *Intestinibacter*, is closely associated with the intestinal metabolic environment, consistent with the findings of previous research (Zhang et al., 2023; Tan et al., 2025). Studies have shown that changes in *Intestinibacter* abundance are correlated with the effects of specific dietary interventions, such as novel ketogenic diet (Meeusen et al., 2025). In the context of this study, MIDCABG patients often present with specific metabolic states (e.g., insulin resistance, chronic inflammation) before surgery, which may provide a niche for the proliferation of *Intestinibacter*. The increased abundance may lead to abnormal bile acid metabolism, which serves as a critical link between the gut, liver, and systemic inflammation and metabolic disorders (Cheng et al., 2025). This further amplifies the risk of POD through the “gut-liver-brain axis” (Shi et al., 2025).

Alterations in the fungal community represented another major finding of this study. Differential abundance analyses identified *Aspergillus* and *Saccharomyces* as the fungal taxa most strongly associated with POD, whereas *Candida* emerged as a candidate fungal taxon in the co-occurrence network analysis. *Candida* is a common intestinal symbiotic fungus that may exacerbate systemic inflammatory states by disrupting the integrity of the intestinal mucosal barrier, activating host immune responses, such as Th17 cell activation (Conti and Gaffen, 2010). Although *Candida* was not identified as the most significant differentiating species in terms of abundance differences in this study, its established role in promoting intestinal barrier dysfunction and systemic inflammation suggests that it may contribute to biological processes associated with increased susceptibility to POD. Previous studies have demonstrated that overgrowth of *Candida albicans* is associated with lower urinary tract infections and irritable bowel syndrome (IBS) in young women, highlighting the role of fungi in sustaining chronic inflammation and disease susceptibility (Ruța et al., 2025). While direct clinical or experimental research linking the gut mycobiome to POD remains limited, substantial evidence indicates that gut mycobiome communities play a significant role in various neuropsychiatric disorders and cognitive impairments, including schizophrenia and Alzheimer’s disease (Hadrich et al., 2024; Yuan et al., 2024; McGuinness et al., 2024). Therefore, it is reasonable to infer that mycobiome dysbiosis may be associated with increased POD susceptibility in elderly patients undergoing MIDCABG.

The metabolites of these key microorganisms may also influence POD. SCFAs are a class of important metabolites produced by gut bacteria through the fermentation of dietary fiber, offering multiple benefits, including anti-inflammatory effects, maintenance of intestinal barrier integrity, and energy provision. Multiple studies have confirmed that butyrate and other SCFAs are associated with improved metabolism, reduced inflammation, and neuroprotective effects (Meeusen et al., 2025; Cheng et al., 2025; Yu et al., 2026). In this study, changes in the bacterome community structure of Group M were likely accompanied by a reduction in the production of butyrate and other SCFAs, while also leading to the generation of various neuroactive metabolites. Research indicates that gut symbiotic bacteria can produce diverse neuroactive compounds, such as GABA, dopamine, and tyramine, by degrading specific precursors, such as glutamine, glutamate, and tryptophan (Tingler et al., 2026). Although metabolites were not directly measured in this study, the increase in *Clostridium* species in Group M may suggest alterations in the neuroactive metabolic pathways associated with these species. These metabolites could directly or indirectly disrupt the functional balance of the central nervous system (CNS) through mechanisms involving the vagus nerve, blood circulation, or modulation of the immune system. Additionally, changes in the specific microbial composition of the gut microbiota in Group M may affect the balance of neuroactive substances such as serotonin, which are closely associated with mood and cognitive function (Tingler et al., 2026; Bai et al., 2026), thereby altering the neurochemical environment of the brain and exacerbating the development of POD.

In addition to key microbial communities, we identified correlations between certain bacterome species and fungi, indicating mutualistic interactions. For instance, *Bacteroides* showed a positive correlation with *Saccharomyces*, suggesting that an increase in *Bacteroides* populations in the gut may lead to a corresponding rise in *Saccharomyces*. While previous studies have documented such interactions within the gut microbiota (Zhang et al., 2022; Lai et al., 2023), their potential role in POD mechanisms in cardiac surgery patients remains unexplored. Future research should further elucidate these interactions and their synergistic effects on POD development.

Our study has certain limitations. First, as a cross-sectional observational study, we primarily identified associations between gut microbiota characteristics and POD using 16S and ITS sequencing. Still, we could not determine the causal relationship—whether specific dysbiosis increased susceptibility to POD or whether POD-related physiological stress or perioperative factors led to alterations in the microbiota. Currently, there is a lack of metagenomic, metatranscriptomic, and systematic metabolomic data (e.g., from blood or cerebrospinal fluid), so our understanding of the relevant functional pathways and mechanisms remains speculative. Future studies should integrate multi-omics approaches, collect longitudinal samples at preoperative, intraoperative, and postoperative time points, and combine detailed clinical data to comprehensively map the dynamic changes in the “gut-brain axis” during the perioperative period using animal models or intervention studies, thereby identifying the most critical microbes and metabolic pathways. Second, this study was a single-center exploratory investigation with a relatively small sample size, which inevitably limits statistical power and increases the risk of both type I and type II errors. Given the high dimensionality of microbiome data and the limited number of events, some microbial associations identified in this study may be unstable and susceptible to overfitting. Therefore, our findings should be interpreted as hypothesis-generating rather than confirmatory. Future studies involving larger multicenter cohorts and external validation datasets are warranted to verify the robustness and reproducibility of the identified microbial signatures. Third, POD is a multifactorial syndrome influenced by advanced age, frailty, cognitive reserve, cardiovascular comorbidities, perioperative medications, inflammatory responses, and surgical stress. Although no significant differences were observed in the baseline and perioperative variables collected in this study, residual confounding cannot be excluded. Due to the limited number of POD cases, adjustment using multivariable models was statistically inappropriate. Therefore, the observed microbiota-POD associations should be interpreted cautiously until confirmed in larger studies with adequate adjustment for established POD risk factors.

## 5 Conclusion

This study demonstrates an association between preoperative intestinal bacterome/mycobiome dysbiosis and POD in patients undergoing MIDCABG, identifying candidate bacterial (*Clostridium* and *Intestinibacter*) and fungal (*Aspergillus*, *Saccharomyces*, and *Candida*) biomarkers of POD. Our findings suggest that POD patients exhibit a susceptible intestinal microecological state before surgery. Despite its limitations, this work establishes a preliminary foundation for microbiota-based prediction models and novel modulation strategies, potentially advancing peri-anesthetic medicine toward more precise and comprehensive approaches.

## Data Availability

The datasets presented in this study can be found in online repositories. The names of the repository/repositories and accession number(s) can be found at: https://www.ncbi.nlm.nih.gov/, PRJNA1452274.
